# Intracranial pressure waveform in patients with essential hypertension

**DOI:** 10.3389/fcvm.2023.1288080

**Published:** 2023-11-21

**Authors:** Matheus Martins da Costa, Ana Luiza Lima Sousa, Mikaelle Costa Correia, Sayuri Inuzuka, Thiago Oliveira Costa, Priscila Valverde O. Vitorino, Polyana Vulcano de Toledo Piza, Gustavo Frigieri, Antonio Coca, Weimar Kunz Sebba Barroso

**Affiliations:** ^1^ Hypertension League—Cardiovascular Section and Health Sciences Post Graduation Program, Federal University of Goias, Goiânia, Brazil; ^2^ Department of Research, Pontifical Catholic University of Goias, Goiânia, Brazil; ^3^ Department of Cardiology, Hospital Israelita Albert Einstein, Goiânia, Brazil; ^4^ Medical Investigation Laboratory 62, University of São Paulo, School of Medicine, Braincare Desenvolvimento e Inovação Tecnológica S.A. São Paulo, Brazil; ^5^ Hypertension and Vascular Risk Unit, Department of Internal Medicine, Hospital Clinic, University of Barcelona, Barcelona, Spain; ^6^ School of Medicine, Clinical Hospital EBSERH, Federal University of Goias, Goiânia, Brazil

**Keywords:** hypertension, intracranial pressure, brain vascular disorders, cerebrovascular diseases, cognitive disfunction

## Abstract

**Background:**

There is a strong association between hypertension and cerebrovascular diseases, but most of the mechanistic bases to justify this correlation remains misunderstood.

**Objective:**

To evaluate intracranial pressure waveform in long-term essential hypertensive patients with a non-invasive device, brain4care (b4c).

**Methods:**

Cross-sectional study in patients with hypertension. Office blood pressure was measured with an automatic oscillometric device. Intracranial pressure evaluation was acquired through a strain sensor that could detect and monitor nanometric skull bone displacements for each cardiac cycle. Under normal physiological conditions, P1 is greater than P2, and the normal P2/P1 ratio is <1. Time to peak (TTP) is the measurement in seconds of the beginning of waveform inscription until P1 and normal values are <0.20 s. The cut-off points ≥1.2 and ≥0.25 s were used to define intracranial hypertension (ICHT).

**Results:**

391 consecutive patients were evaluated (75% female, mean age 64.3 ± 12.0 years). Mean value of P2/P1 ratio was 1.18 ± 0.25 and TTP 0.18 ± 0.63 s The obtained P2/P1 ratios were divided in three categories according to results of previous studies of normalcy (<1.0), intracranial compliance disturbance (1.0–1.19) and ICHT (≥1.2). Normal intracranial pressure was observed in 21.7% of patients, intracranial compliance disturbance in 32.7% and intracranial hypertension in 45.6%. Females showed a higher prevalence of ICHT (50.3%).

**Conclusion:**

The prevalence of 45.6% intra-cranial hypertension in patients with long-term hypertension, particularly in women, and in those over 65 years old, emphasizes the importance of evaluate intracranial pressure behaviour in these patients and raise a question concerning the real ability of cerebral autoregulation and vascular barriers to protect the brain.

## GRAPHICAL ABSTRACT

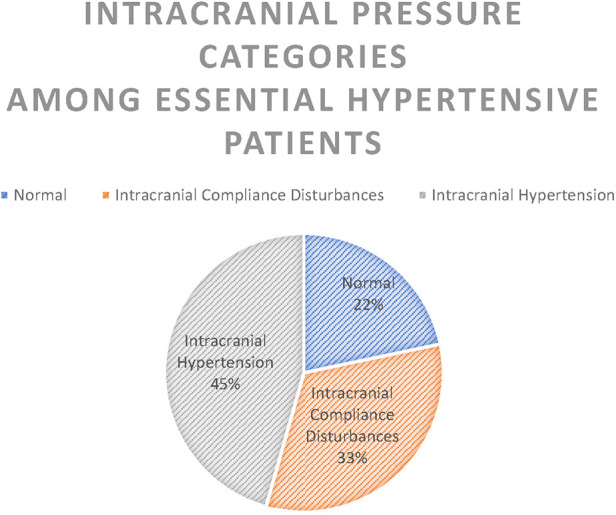

## Introduction

According to World Health Organization (WHO), hypertension (HT) remains as the leading cause of death around the globe and the absolute number of hypertensive adults has doubled in the last three decades (1).

There is a strong association between HT and cerebrovascular diseases, particularly with stroke and cognitive impairment. Despite of that, most of the mechanistic bases to justify this correlation remains to be established. Clearly there is a structural and functional damage in arterial bed concomitant with a pathological increase in blood pressure (BP) but, at least by the concept of cerebral autoregulation and vascular brain barrier, tissues and vessels inside the skull should be safe in early phases of HT (2–4).

Using a minimally invasive system to monitor intracranial pressure (ICP), Mascarenhas et al. (5) showed in 2012 that Monro-Kellie doctrine of an inextensible skull after closure of the fontanels is not completely true. Since then, the non-invasive device brain4care (b4c) have been validated for monitoring intracranial pressure waveform, enabling wider use of this methodology in clinical practice (6–9).

More recently, in 2021, Fernandes et al. (10) reported that after three weeks of induced renovascular hypertension in a rat model, there was a deleterious effect on ICP dynamics compatible with intracranial hypertension.

There is a grey zone concerning ICP behaviour in chronic hypertensive patients, and very little is known on this subject. To our knowledge, this is the first report evaluating intracranial pressure behaviour in essential hypertensive patients, with the potential to bring some light to this dark side of human history concerning cerebrovascular and cognitive disorders.

## Patients and methods

### Selection of patients

Since November 2022 to May 2023 all adult patients seen in the Research Center for Cardiometabolic Diseases of the Hypertension Unit, Federal University of Goias, Brazil, and scheduled to perform ambulatory (ABPM) or home blood pressure measurement (HBPM) were invited to participate in the study. The study was designed as part of a cross-sectional analysis approved by the Ethic Committee, Clinical Hospital—Federal University of Goias, number 70448823.1.0000.5078 with the main objective to evaluate the behaviour of non-invasive ICP waveform in patients with sustained hypertension.

After a routine medical history and physical examination, the following parameters were obtained: age, sex, race, body mass index (BMI) in Kg/ m^2^, office systolic (SBP) and diastolic (DBP) blood pressure in mmHg, previous history of any cardiovascular (CV) clinical disease, myocardial infarction (MI), stroke, dyslipidemia, diabetes (DM), length of HT diagnosis in years and number and class of anti-hypertensive drugs.

All these variables were collected the same day when intracranial pressure waveform was measured and were managed by using the REDCap electronic data capture tool hosted in Federal University of Goias.

### Blood pressure measurements

Office blood pressure was measured according to the methodology recommended by the Brazilian Guidelines on Hypertension (11) with an automatic oscillometric device (Omron HBP-1100). Three consecutive measurements were taken after 5 min of rest, with the patient in a sitting position, and the average of the last two measurements was recorded. By guidelines convention, controlled HT was defined when both SBP and DBP were under 140 mmHg and 90 mmHg.

### Assessment of intracranial pressure

**Figure 1:**
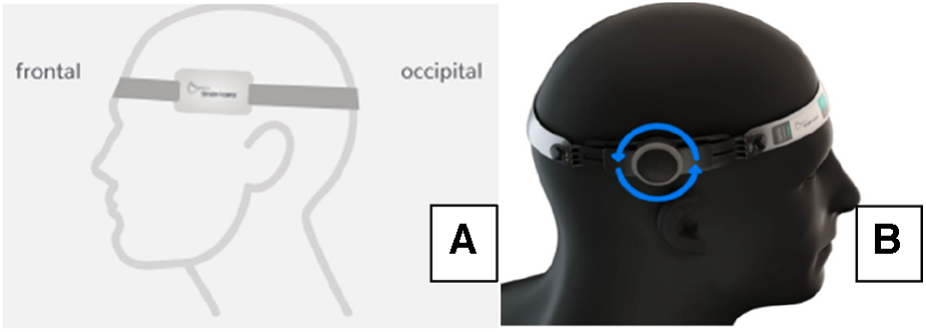
(**A**) Sensor placement; (**B**) rack adjustment.

Intracranial pressure evaluation was performed in a private and silent room with patients at lying position and monitoring waveforms for seven minutes. The first and last minutes were discarded. The non-invasive device brain4care sensor was positioned on the patient's scalp and the morphology of the ICP waves was acquired through a strain sensor that could detect and monitor nanometric skull bone displacements each cardiac cycle (Figure 1).

**Figure 2:**
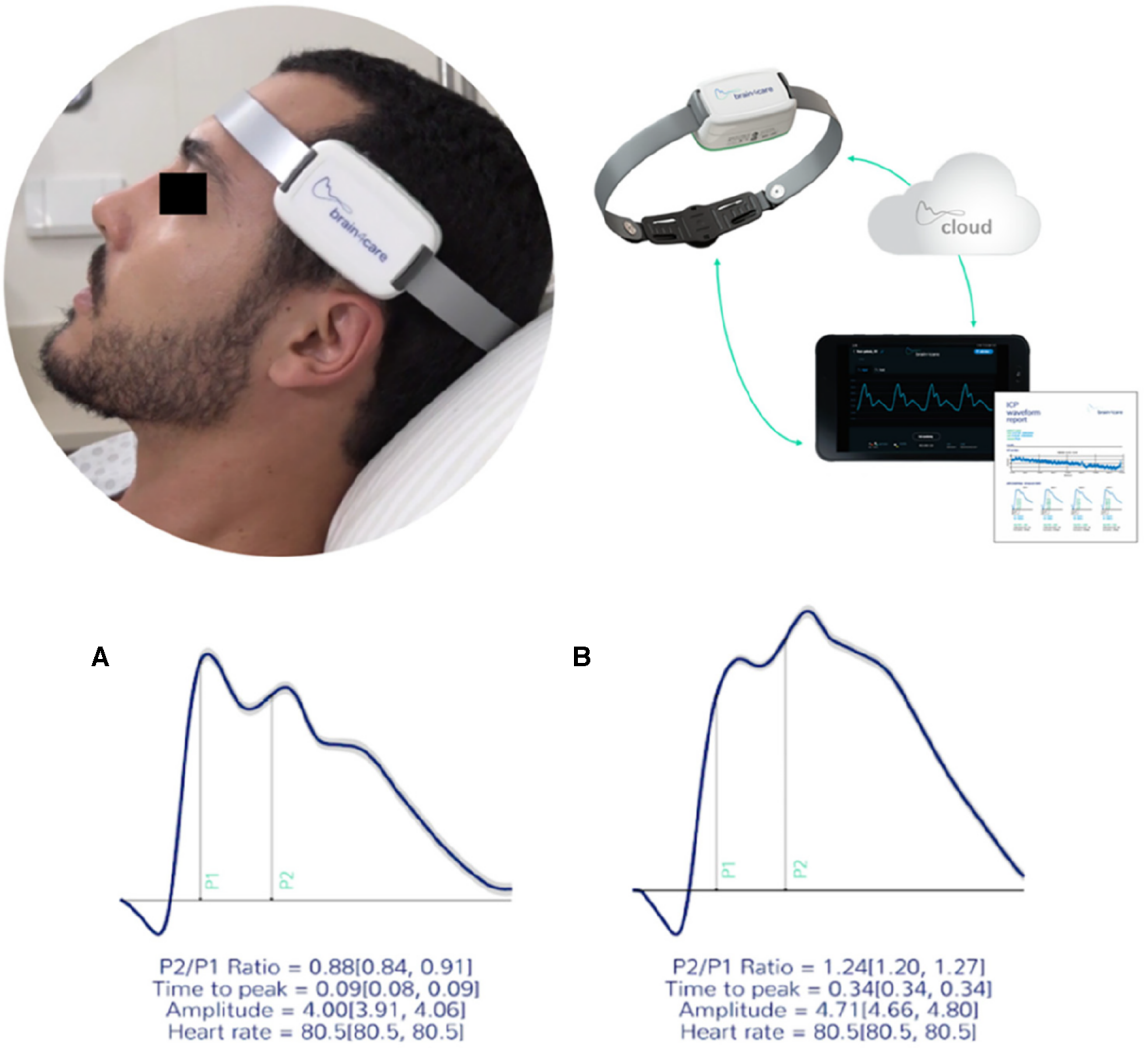
The b4c noninvasively ICP waveform real time monitoring. All data collected is immediately processed by the b4c system analytical software resulting in the quantitative and qualitative reports. (**A**) waveforms depicting normal standards (P2/P1 < 1.2 and TTT < 0.25 seg) and (**B**) altered standards (P2/P1 ≥ 1.2 and TTP ≥ 0.25 seg). (6).

The ICP waveform has two distinct amplitude peaks: P1 and P2. The first P1 amplitude results from transmission of systolic cerebral blood flow and P2 amplitude is associated with brain compliance to intracranial pressure (Figure 2).

Under normal physiological conditions, P1 is greater than P2, and the normal P2/P1 ratio is <1. Time to peak (TTP) is the measurement in seconds of the beginning of waveform inscription until P1 and normal values are <0.20 s. The waveforms and data obtained during non-invasive ICP monitoring were automatically digitized, filtered and amplified by the device. Although intracranial hypertension (ICHT) is usually defined as a sustained (>5 min) intracranial pressure over 20 mmHg (12), using the b4c non-invasive evaluation the cut-off point identified to define ICHT by P2/P1 ratio was ≥1.2 and the cut-off for time to peak (TTP) ≥0.25 s. The values of P2/P1 from 1.0–1.19 and the TTP values from 0.20–0.24 s were considered as a grey zone of abnormal intracranial compliance but not ICHT (13–17).

### Statistical analysis

Data were first recorded on the REDCap platform and exported in an excel spreadsheet format. The database was organized and cleaned, removing duplicates, and then exported to SPSS IBM version 26.0 to proceed with statistical analysis. Categorical variables were presented with their frequencies and proportions. The analysis of categorical data associations was performed using the chi-square test. Quantitative variables with data of a continuous nature were firstly analysed in terms of distribution, applying the Kolmogorov-Smirnov test; the presentation of these data was done with mean values ​​and standard deviation when normally distributed. For the analysis of these data, parametric tests were applied, when applicable, such as the T-student test or Analysis of Variance (ANOVA).

## Results

Non-invasive b4c ICP waveform was performed in 401 patients with essential long-term hypertension evaluated during a period of seven months. Ten patients were excluded due to repeated exams or inadequate quality of the signal, thus the final sample included 391 patients. The mean age was 64.3 ± 12.0 years, 75% were female, and mean BMI was 29.8 ± 6.3 Kg/m^2^. The average of time since the hypertension diagnosis was 20.0 ± 12.8 years. Sociodemographic, anthropometric data and clinical variables are shown in Table 1.

**Table 1:** Patient's characterization according to sociodemographic, anthropometric, and clinical variables (*n* = 391).

Variable	*n* (%)
Sex	* *
Female	292 (74.7)
Male	99 (25.3)
Body Mass Index (BMI) Kg/m^2^	* *
≤18.5	3 (0.8)
18.6–25.0	80 (20.5)
25.0–29.9	138 (35.3)
≥30.0	170 (43.5)
Age group	*** ***
≥65 years	219 (56.0)
<65 years	172 (44.0)
Diabetes mellitus	*** ***
Yes	161 (41.2)
No	230 (58.8)
Myocardial infarction	*** ***
Yes	43 (11.0)
No	348 (89.0)
Stroke	*** ***
Yes	29 (7.4)
No	362 (92.6)
Dyslipidaemia	*** ***
Yes	312 (79.8)
No	79 (20.2)
Controlled hypertension	*** ***
Yes No	205 (52.4) 186 (47.6)
Length of HT diagnosis (years)	** **
<15 years	183 (46.8)
15–30 years	120 (30.7)
≥30 years	88 (22.5)
Number of anti-hypertensive drugs	
1 2 or 3 >3	101 (25.8) 232 (59.3) 58 (14.8)

Mean value of P2/P1 ratio of all cohort was 1.18 ± 0.25 and TTP 0.18 ± 0.63 s. The evaluation of P2/P1 and TTP behaviours in the different clinical settings as well as in the sociodemographic and anthropometric situations, did not find differences concerning P2/P1 ratio; however, TTP showed significant increased values in patients over 65 years and in those with obesity (Table 2).

**Table 2:** Distribution of means and standard deviations of P2/P1 ratio and TTP according to sociodemographic and clinical variables (*n* = 391).

Variable	P2/P1	* *	TTP (s)	*p*-value
* *	Mean (±SD)	* *	Mean (±SD)	* *
Sex	*** ***	*** ***	*** ***	*** ***
Male	1.128 (0.229)	0.370*	0.167 (0.046)	0.467*
Female	1.192 (0.250)	*** ***	0.191 (0.067)	*** ***
Age (≥65 years)	*** ***	*** ***	*** ***	*** ***
Yes	1.151 (0.242)	0.875*	**0.207** (**0.080)**	**0**.001*
No	1.209 (0.249)	*** ***	0.167 (0.037)	*** ***
Stroke	*** ***	*** ***	*** ***	*** ***
Yes	1.189 (0.262)	0.267*	0.173 (0.491)	0.765*
No	1.175 (0.246)	*** ***	0.186 (0.064)	*** ***
Diabetes mellitus			** **	** **
Yes No	1.163 (0.253) 1.186 (0.242)	0.899*	0.188 (0.075) 0.183 (0.053)	0.635*
Dyslipidemia				
Yes No	0.173 (0.243) 0.188 (0.260)	0.281*	0.184 (0.058) 0.185 (0.064)	0.378*
BMI categories		0.119**		**0**.004*
18.6–25.0 25.0–29.9 ≥30.0	1.128 (0.249) 1.194 (0.261) 1.189 (0.231)		0.170 (0.044) 0.180 (0.047) **0.196** (**0.079)**	** **
Controlled HT				** **
Yes	1.174 (0.240)	0.735*	0.183 (0.051)	0.384*
No	1.179 (0.254)	*** ***	0.187 (0.074)	*** ***
Length of HT diagnosis	** **	0.528**	*** ***	0.536**
<15 years	1.191 (0.253)	*** ***	0.187 (0.055)	*** ***
15–30 years	1.167 (0.232)	*** ***	0.186 (0.080)	*** ***
≥30 years	1.159 (0.252)	*** ***	0.178 (0.049)	*** ***
≥30.0	1.189 (0.231)		0.196 (0.079)	
Number of anti-hypertensive drugs		0.149**		0.339**
1 2–3 >3	1.140 (0.237) 1.200 (0.252) 1.151 (0.236)	*** ***	0.177 (0.052) 0.187 (0.053) 0.190 (0.105)	*** ***

P2/P1 ratio: ratio between P1 amplitude (resulting from transmission of systolic cerebral blood flow) and P2 amplitude (associated with brain compliance to intracranial pressure).

TTP: measurement in seconds of the beginning of waveform inscription until P1.

* *t*-student test.

** ANOVA.

The obtained P2/P1 ratios were divided in three categories according to the results of previous studies of normalcy (<1.0), intracranial compliance disturbance (1.0–1.19) and ICHT (≥1.2). A normal intracranial pressure was observed in 21.7% of all patients, 32.7% exhibited intracranial compliance disturbance, and intracranial pressure was observed in 45.6%. Females showed a significant higher prevalence of ICHT (50.3%). When comparing the scenarios of P2/P1 ≥1.2 and P2/P1 <1.2 females also showed a significant higher prevalence of ICHT (Table 3).

**Table 3:** Bivariate analysis of the P1/P2 ratio according to sociodemographic and clinical variables (*n* = 391).

	P2/P1		
	<1.2	≥1.2	*p*-value
	*n* (%)	*n* (%)	
Sex	*** ***	*** ***	**0**.**002**
Female	145 (68.4)	**147** (**82.1)**	* *
Male	67 (31.6)	32 (17.9)	*** ***
Age group	*** ***	*** ***	0.058
Adults	84 (39.6)	88 (49.2)	* *
Elderly	128 (60.4)	91 (50.8)	*** ***
Stroke	*** ***	*** ***	0.504
No	198 (93.4)	164 (91.6)	*** ***
Yes	14 (6.6)	15 (8.4)	*** ***
Diabetes	*** ***	*** ***	0.577
No	122 (53.0)	108 (60.3)	*** ***
Yes	90 (42.5)	71 (38.7)	*** ***
Dislipidemia	*** ***	*** ***	0.966
No	43 (20.3)	36 (20.1)	*** ***
Yes	169 (79.7)	143 (79.9)	*** ***
Myocardial infarction	*** ***	*** ***	0.919
No	189 (89.2)	159 (88.8)	*** ***
Yes	23 (10.8)	20 (11.2)	*** ***
BMI (Kg/m^2^)	*** ***	*** ***	0.136
18.5–24.9	51 (24.4)	29 (16.2)	*** ***
25.0–30.0	70 (33.5)	68 (38.0)	*** ***
≥30.0	88 (42.1)	82 (45.8)	*** ***
Lenght of HT diagnosis	*** ***	*** ***	0.418
<15 years	93 (43.9)	90 (50.3)	*** ***
15–30 years	70 (33.0)	50 (27.9)	*** ***
≥30 years	49 (23.1)	39 (21.8)	*** ***
Controlled HT	*** ***	*** ***	0.707
Yes	113 (53.3)	92 (51.4)	*** ***
No	99 (46.7)	87 (48.6)	
Number of anti-hypertensive drugs			0.061
1 2–3 >3	64 (30.2) 11 (54.2) 33 (15.6)	37 (20.7) 117 (65.4) 25 (14.0)	

P2/P1 ratio: ration between P1 amplitude (resulting from transmission of systolic cerebral blood flow) and P2 amplitude (associated with brain compliance to intracranial pressure) transferred to the b4c system analytical software.

Chi square test.

## Discussion

The sample in our study was composed by adult patients with long-term essential hypertension with high prevalence of cardiovascular (CV) risk factors such as diabetes (41.2%) and dyslipidaemia (79.8%), and previous CV disease such as MI (11.0%) and stroke (7.4%). In addition, most of them were aged over 65 years and had overweight and obesity (Table 1). Despite the very well stablished association between HT and cerebrovascular diseases (ischaemic and haemorrhagic stroke) and cognitive impairment, the behaviour of intracranial pressure in these populations have not been investigated so far due to the absence of validated non-invasive methodologies. Fortunately, the validation of the non-invasive brain4care device for this purpose permits a safe and precise intracranial pressure assessment, and the investigation of the effect of short and long-term systemic hypertension on intracranial pressure waveform, contributing to a better understanding of the pathophysiology of brain damage induced by high blood pressure (5, 6).

Disturbances in blood flow delivery to the brain and blood-brain barrier (BBB) permeability seems to occur before neurodegeneration (18, 19). Disruption of BBB can occur in certain medical conditions, such as infections, inflammation or injury. When the BBB becomes compromised, it can lead to increased permeability and allow harmful substances to enter the brain that would normally be restricted to pass through. These aspects reinforce the importance of a better understanding of the role of intracranial pressure in hypertension since fluid retention, endothelial dysfunction and remodelling, of either extracranial or intracranial cerebral arteries, particularly of penetrating small vessels into the white matter, plays an important role in the pathophysiology of brain disturbances induced by changes in blood pressure (20).

We found an average value of P2/P1 ratio of 1.18 ± 0.25, and a TTP mean value of 0.18 ± 0.63 s in the whole sample of patients with hypertension, and the prevalence of P2/P1 >1.0 defining intracerebral hypertension was 78.3%. Even when using a stricter cut-off point P2/P1 ≥1.2 for ICHT the prevalence was 45.6%. The fact that at least half of the patients with long-term hypertension showed abnormal values of intracranial pressure deserves attention concerning the possibility of a lost in the capacity of cerebral autoregulation and BBB to protect brain tissue in hypertension. In opposition to what was believed, a previous publication in animal models revealed an increase in intracranial pressure just a few weeks after inducing renovascular hypertension, meaning that this injury begins in the early phases of BP elevation. The observed results in hypertensive rats and in patients with essential hypertension suggests that several concepts regarding cerebral autoregulation should be revisited (10, 21).

When we look for differences in the mean values of P2/P1 ratio and TTP in the recorded sociodemographic, anthropometric, and clinical variables (Table 2), the only differences observed concerned a greater TTP in elderly patients and also in those with obesity. Considering that these differences would become apparent from the very beginning of HT, at least in animal models, perhaps a long-term exposition to high blood pressure values over years in our patients could be responsible for an important damage in the neuroprotective mechanisms (21–23). Comparing the scenarios of P2/P1 ≥1.2 or <1.2 we only found a higher prevalence of ICHT in females. Taking into consideration that stroke and dementia are more frequent in women worldwide, we can speculate that perhaps these differences in the autoregulatory capacity of intracranial pressure and BBB permeability in women, may play an important role in the pathophysiology of brain damage, although further research in this point is guaranteed (22, 24). Interestingly, P2/P1 behaviour equivalent to ICHT did not differ when comparing patients with controlled or uncontrolled hypertension, neither with the number and classes of antihypertensive drugs, highlighting once again the possibility of a loss of ability by the brain to regulate higher and lower BP ranges even under treatment with blood pressure lowering drugs (3).

Our study has some limitations; It is important to note that it is a cross-sectional analysis of the cohort, and the data related to the behaviour of P2/P1 and TTP in different subsets of patients with hypertension regarding anthropometric, sociodemographic and comorbidities may express some differences in a larger sample of subjects and, of course, that longitudinal studies are needed to evaluate other features in a cause-and-effect relationship. The strengthen of this study is to show by the first time a huge prevalence of intracranial hypertension in patients with essential hypertension and long-term exposure to high blood pressure values.

In conclusion, the observation of a prevalence of 45.6% of P2/P1 ratio over 1.2 equivalent to intracranial hypertension in patients with long-term hypertension, particularly in women, and in those over 65 years old emphasizes the importance of closely evaluate non-invasive intracranial pressure waveform in hypertensive patients. The strategy to maintaining optimal systolic blood pressure levels between 120 and 140 mmHg may help to reduce stress on blood vessels, minimizing the risk of BBB disruption. Our findings also raise a question about the accepted concept about the capacity and effectiveness of cerebral autoregulation and vascular barriers to protect the brain in the context of blood pressure elevations. This understanding can lead to a potential therapeutic avenue for hypertension-related brain complications.

## Data Availability

The raw data supporting the conclusions of this article will be made available by the authors, without undue reservation.
